# The BDNF Val66Met polymorphism moderates the relationship between cognitive reserve and executive function

**DOI:** 10.1038/tp.2015.82

**Published:** 2015-06-30

**Authors:** D D Ward, M J Summers, N L Saunders, K Ritchie, J J Summers, J C Vickers

**Affiliations:** 1 School of Medicine, University of Tasmania, Hobart, TAS, Australia; 2 Wicking Dementia Research and Education Centre, University of Tasmania, Hobart, TAS, Australia; 3 School of Social Sciences, University of the Sunshine Coast, Sippy Downs, QLD, Australia; 4 Neuroepidemiology of Ageing Research Unit, Imperial College, London, UK; 5 Inserm, U1061 Neuropsychiatry, Montpellier, France; 6 Research Institute for Sport and Exercise Sciences, Liverpool John Moores University, Liverpool, UK

## Abstract

The concept of cognitive reserve (CR) has been proposed to account for observed discrepancies between pathology and its clinical manifestation due to underlying differences in brain structure and function. In 433 healthy older adults participating in the Tasmanian Healthy Brain Project, we investigated whether common polymorphic variations in apolipoprotein E (*APOE*) or brain-derived neurotrophic factor (*BDNF)* influenced the association between CR contributors and cognitive function in older adults. We show that *BDNF* Val66Met moderates the association between CR and executive function. CR accounted for 8.5% of the variance in executive function in *BDNF* Val homozygotes, but CR was a nonsignificant predictor in *BDNF* Met carriers. *APOE* polymorphisms were not linked to the influence of CR on cognitive function. This result implicates *BDNF* in having an important role in capacity for building or accessing CR.

## Introduction

Observations of significant heterogeneity in the clinical manifestation of underlying brain pathology has led to the development of the concept of cognitive reserve (CR).^1^ CR level is considered to be determined by both biological and environmental exposures, notably intelligence,^2^ education^3^ and occupational attainment.^4^ Epidemiological evidence further supports the notion of CR by demonstrating consistent associations between lifestyle characterized by intellectual and social engagement and slower cognitive decline.^5^ In older persons, functional magnetic resonance imaging studies of neural networks suggest that differences in functionally connected regions in persons with high CR may enhance the compensatory capacity of individuals in the face of both normal and pathological brain aging.^6^ Alzheimer's disease (AD) patients with higher educational and occupational attainment have more rapid cognitive decline than those with lower attainment, consistent with the idea that at a given common level of severity, underlying AD pathology is more advanced in patients with more CR.^7^ That individuals with higher CR display greater resistance to the effects of neuropathology is likely due to induced cortical plasticity caused by a prolonged mismatch between functional supply and task demands,^8^ resulting in more flexible and denser neural networks.^9^ As adult cognitive function and cognitive aging show significant heritability,^10^ research examining the interaction between genes and CR proxies is justified. The most likely candidate genes for CR are those that interact with environmental factors to induce effects on cognitive functions.^11^

One gene that potentially interacts with CR is apolipoprotein E (*APOE*). Although it has been established that specific allelic variants in the *APOE* gene are associated with risk for late-onset AD,^12^ it is less clear whether other allelic variants of the *APOE* gene impart healthy cognitive function.^13, 14^ However, *APOE* polymorphisms have been shown to exert divergent neuroprotective effects.^15, 16^ For example, the presence of *APOE* ɛ4 is associated with an increased rate of AD-related hippocampal atrophy^17^ and impaired synaptic plasticity^18^ when compared with *APOE* ɛ4 noncarriers. Lifestyle activities that require cognitive effort (for example, completing puzzles and playing chess) predict verbal fluency and semantic memory recall in *APOE* ɛ4 noncarriers but not in carriers.^19^

A second gene that may be associated with CR is brain-derived neurotrophic factor (*BDNF*). The encoded neurotrophin is crucial to neuronal survival, maintenance, neurogenesis and synaptic plasticity.^20, 21^ The *BDNF* Val66Met polymorphism affects activity-dependent secretion of BDNF, with the Met allele associated with reduced depolarization-induced BDNF release into the synapse.^22^ *BDNF* Met alleles have been associated with reduced memory capacity,^22^ but not consistently.^14, 23^ Similar to *APOE*, impaired synaptic plasticity is a feature of *BDNF* Met alleles.^24, 25^

Synaptic plasticity is a key mechanism that facilitates the development of CR.^26^ Research to date has examined CR in the presence of pathology; however, as CR is developmentally acquired through both intrinsic (biologic) factors such as genetic heritability as well as extrinsic (environmental) factors such as education, it is imperative to examine the development of CR prior to the onset of clinical symptomatology. We aimed to use baseline data from the Tasmanian Healthy Brain Project (THBP)^27^ to investigate the relationship between CR, cognitive function and *APOE* and *BDNF* Val66Met polymorphisms in healthy older adults (50–79 years). We tested two hypotheses. First, that higher CR is associated with better cognitive performance. Second, that *BDNF* Val66Met and *APOE* polymorphisms independently moderate the association between CR and cognitive performance, with carriers of the putative detrimental alleles (Met and ɛ4, respectively) showing a reduced influence of CR when compared with noncarriers.

## Materials and methods

### Participants

The participants were 433 community-residing healthy older adults, aged 50–79 years, who were all native-English speakers or spoke English as a primary language. They had consented to participate in the THBP, which involves comprehensive annual assessments of neuropsychological, health and psychosocial function. The THBP is a prospective longitudinal study examining whether late-life education provides protection from dementia through enhancement of CR.^27^ Subjects were excluded from participating if they had a history of any condition independently associated with impairments to cognitive function (dementia; multiple sclerosis; previous significant head injury requiring hospitalization; epilepsy; history of cerebrovascular complications; diabetes—poorly controlled; blood pressure complications—poorly controlled; other neurological disorders; chronic obstructive pulmonary disease; heart disease; blindness; deafness; psychiatric disorder). The THBP was conducted with full approval from the Tasmania Human Research Ethics Network and in accordance with the ethical guidelines of the National Health and Medical Research Council of Australia.

### Materials

A comprehensive test battery was completed by subjects as described in detail elsewhere.^27^ Multiple objective measures were used to screen participants for: symptoms of dementia, clinically significant symptoms of depression or anxiety, general health, medical conditions, prescription medication use, drug and alcohol use, handedness, height, weight, marital status, educational and occupational history.

### Neuropsychological assessment battery

Cognitive functions were assessed using tests that measured learning and memory, working memory, executive function and language processing, and are described in detail elsewhere.^27^ The test battery incorporated standardized neuropsychological measures with established reliability and validity in the measurement of: visual and verbal episodic memory, visual and verbal immediate memory span, visual and verbal working-memory capacity, word knowledge, semantic memory recall, language comprehension and multiple executive functions including attention, concentration, information-processing speed, decision-making capacity and reaction time.

### Assessment of CR

To generate an estimate of CR, we used a previously developed equation to combine measures of lifetime education, occupational attainment, intelligence and participation in cognitively stimulating leisure activities into a single variable.^28^ The Wechsler Test of Adult Reading^29^ was used to estimate premorbid intellectual capacity, the Lifetime of Experiences Questionnaire^30^ to quantify prior lifetime participation in cognitive activities and the Medical Health Screening questionnaire to record the number of years of prior formal education.^27^ Briefly, factor analysis (principal components extraction method) with Varimax rotation combined seven CR proxy variables into four significant latent factors. In combination, these factors explained 77.10% of the variance in the data set. *Z*-scores for each component were generated using the regression method, with an estimate of overall CR resulting from the addition of each factor score into the total score.

### Genotyping

DNA samples were collected with Oragene DNA self-collection kits (Genotek, ON, Canada, 2012). *APOE* and *BDNF* genotypes were determined through one-step amplified refractory mutation system PCR^31^ and subsequent gel electrophoresis. For *APOE*, rs429358 and rs7412 were determined by following the method described by Donohoe *et al.*^32^ For *BDNF*, Val66Met was determined by following the method described by Sheikha *et al.*^33^ PCR amplifications were undertaken in a 12-μl reaction volume that contained ~50 ng of genomic DNA. PCR amplicons were resolved on 2% agarose gel. Genotyping was repeated on samples to ensure accuracy.

### Procedure

Trained assessors carried out the neuropsychological testing as part of the baseline THBP assessments.^27^ The assessment process took ~4 h to complete and was undertaken in a quiet room. Subjects were encouraged to take 10-min breaks when required to reduce fatigue.

### Data analysis

Prior to the main analyses, variables of CR and cognitive function were generated. To represent CR, we used a previously developed equation to create a comprehensive single-point measure of the construct.^28^ Standardized scores of CR variables (Table 1) were entered into the CR equation to yield total CR for each subject. Composite cognitive domain variables were also computed through a principal components analysis that combined measured variables (raw scores) into specific domains (episodic memory, working memory, executive function and language processing). The extracted factor that explained the highest proportion of variance was retained to represent each cognitive domain (Table 2). The *APOE* and *BDNF* predictor variables were coded as carriers or noncarriers of the detrimental allele, ɛ4 and Met, respectively. To assist in interpretation, subjects with the *APOE* ɛ2/ɛ4 polymorphism were excluded from the analysis.

The primary analyses used PROCESS v2.11 (ref. 34) to test whether CR was associated with cognitive function, either independently or through CR × *APOE*/CR × *BDNF* interaction. PROCESS is a computational tool for path analysis-based moderation and mediation analysis that provides coefficient estimates for total, direct and indirect effects of variables using ordinary least squares regression. Prior to analysis, the continuous independent predictor (CR) was mean-centered to reduce potential multicollinearity. Subsequent analyses involved testing for direct effects of predictors (CR, *APOE* and *BDNF*) and indirect effects of possible CR moderators (*APOE* and *BDNF*) on cognitive function domains while covarying for age and gender. First, regression equations were fitted to cognitive domain data and the predictive capacity of age, CR and gender were assessed. Second, *APOE*/*BDNF* data and corresponding CR-gene product variables were entered to test for CR moderation in separate models. An alpha value of 0.05 was used for all statistical tests, and all data were analysed in SPSS v21 (IBM, Armonk, NY, USA).

## Results

### Participants

The sample comprised 433 participants with a mean age of 62.16 years (s.d.=6.81) and an above-average estimated full-scale intelligence quotient (*M*=112.47, s.d.=5.52). Participants were mostly female (66.7%) and had completed an average of 13.97 (s.d.=2.73) years of formal education. To assist in interpretation, 11 participants were excluded due to possessing the *APOE* ɛ2/ɛ4 genotype. The characteristics of the remaining participants are presented in Table 1. The *APOE* (*χ*^2^_(1,*N*=422)_=0.02, *P*=0.90) and *BDNF* Val66Met (*χ*^2^_(1,*N*=422)_=0.31, *P*=0.58) genotype distributions did not differ significantly from the Hardy–Weinberg equilibrium.

### CR, age and gender

PROCESS was used to fit linear regression models to the cognitive domain data. With the age, CR and gender predictors entered, significant models were produced for all assessed cognitive functions: episodic memory (*F*_(3,397)_=25.53, *P*<0.01, *R*^2^=0.16); working memory (*F*_(3,397)_=19.66, *P*<0.01, *R*^2^=0.13); executive function (*F*_(3,397)_=37.54, *P*<01, *R*^2^=0.22); and language processing (*F*_(3,411)_=23.79, *P*<0.01, *R*^2^=0.15). The CR variable had a significant positive association with each cognitive domain (*P*<0.01), whereas the age variable had a significant negative association with each cognitive domain (*P*<0.01), excluding language processing (*P*=0.62). The gender variable had a significant positive association with episodic memory (*P*<0.01) and executive function (*P*<0.05), whereby females outperformed males. The individual contribution that each predictor made to the model is detailed in Table 3.

### Gene–CR interactions

**Figure 1:**
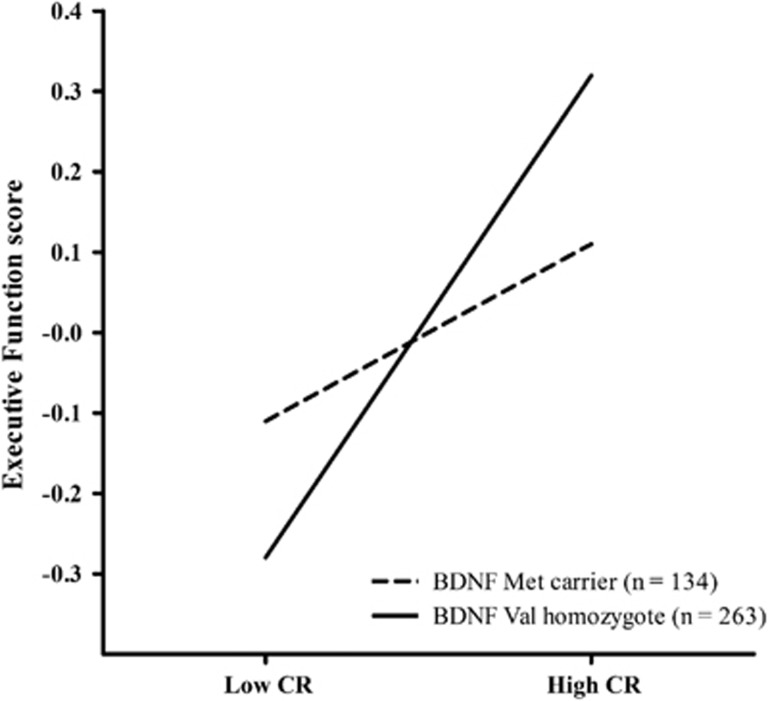
*BDNF* Val66Met moderates the relationship between CR and executive function scores. The plot represents age- and gender-adjusted executive function performance as predicted by the composite CR proxy variable for those with low CR (CR scores less than the mean–1s.d.) and high CR (CR scores greater than the mean+1s.d.) for *BDNF* Met carriers and Val homozygotes, separately. *BDNF*, brain-derived neurotrophic factor; CR, cognitive reserve.

We then assessed whether the inclusion of *APOE*/*BDNF* Val66Met allelic carrier data significantly improved the fit of the models. Notably, no significant main effects of genetic predictors, *APOE* or *BDNF* Val66Met, were identified for any cognitive domain. A moderation analysis (PROCESS) was then conducted examining whether the inclusion of CR × *APOE* or CR × *BDNF* interaction terms significantly improved the fit of regression models. Results indicated that a single genetically based moderation effect on CR was present (Table 3). Specifically, inclusion of the CR × *BDNF* Val66Met interaction term led to a significant increase in the amount of variance in executive function explained by the model (Δ*R*^2^=0.01, *P*=0.05). Simple slopes analysis was conducted in order to determine the basis of the moderation effect of *BDNF* polymorphism on the conditional effect between CR and executive function, and to determine whether the slopes statistically differed from zero for *BDNF* Met carriers and noncarriers, separately. These analyses indicate that a significant positive relationship between CR and executive function was identified in *BDNF* Val homozygotes (*β*=0.13, *t*=5.56, s.e.=0.02, *P*<0.01), but was not evident in *BDNF* Met carriers (*β*=0.05, *t*=1.52, s.e.=0.03, *P*=0.13). In *BDNF* Val homozygotes, CR accounted for a significant 8.6% of variance in executive function performance. In *BDNF* Met carriers, CR accounted for a nonsignificant 1.5% of variance in executive function performance. Simple slopes are presented in Figure 1.

## Discussion

The present study was designed to investigate whether a composite measure of CR was associated with healthy cognitive function, either independently or through an interaction with genetic *APOE*/*BDNF* Val66Met polymorphic data, in participants of the THBP. The first hypothesis, that higher CR is associated with better cognitive performance, was supported. Our sample showed significant positive relationships between CR and cognitive function across all cognitive domains, after accounting for the effects of age and gender. The second hypothesis, that *BDNF* Met carriers and *APOE* ɛ4 carriers would display a weaker relationship between CR and cognitive function when compared with their respective noncarriers, was partially supported. The analyses revealed a single significant CR × *BDNF* interaction in predicting age- and gender-adjusted executive function performance. In this regard, the relationship between CR and executive function was moderated by *BDNF* genotype, with a positive association between the variables present in *BDNF* Val homozygotes that was absent in *BDNF* Met carriers.

CR is of interest due to its potential role in varying the age at which an individual develops dementia and the subsequent rate of dementia progression experienced,^9^ as well as mitigating against the effect of other central nervous system insults. However, although previous research frequently highlights the relationship between increased CR and reduced dementia incidence,^35^ other investigations have suggested that CR is also involved in nonpathological cognitive function. For instance, during a memory task, higher functional connectivity, indicative of heightened cognitive effort, is found in subjects with lower CR, despite achieving the same level of performance as those with higher CR.^36^ Similarly, CR was positively associated with brain volume but negatively associated with cortical activity during a visual encoding task.^37^ Such findings are consistent with the CR hypothesis, which posits that CR is implicated in nonpathological cognitive function,^1^ particularly in relation to cerebral network efficiency.^38^ Our finding, that CR was positively associated with all four assessed cognitive domains, adds further evidence that CR accounts for healthy cognitive function and that cognitive associations with CR are not limited to protection from the clinical expression of pathological processes. At the very least, it may be that higher scores on CR proxy variables would result in a delayed onset of dementia due to the pre-existing cognitive advantage afforded to high-CR individuals through greater education, occupational attainment, intelligence and participation in cognitively stimulating lifestyle activities. In high-CR individuals a greater loss of cognitive function, resulting from greater levels of neuropathology, would be required to produce functional deficits of sufficient severity to meet diagnostic criteria for dementia.

A comprehensive review of the evidence for genetic contributions to CR noted that those genes that interact with environmental factors to produce a cognitive effect are the most likely candidates.^11^ In our study, we identified a significant moderating influence of *BDNF* Val66Met polymorphism on the relationship between CR and executive function. In those individuals who were *BDNF* Val homozygotes, a one-point increase in CR was associated with a 0.13-point increase in executive function. Comparatively, in those individuals who were *BDNF* Met carriers, a one-point increase in CR was associated with a nonsignificant 0.05-point increase in executive function. This finding is of particular importance due to the overlap between CR and executive function.^39^ Cognitive flexibility is suggested to be both one of the processes that mediate the compensatory benefits associated with higher CR^1^ and a mental ability considered as a component of executive function.^40^ In addition, both CR and executive function are associated with frontal lobe activity,^41, 42^ with a positive association of education and frontal engagement present in older adults that is not seen in younger adults.^42^ Clinical studies confirm that there is an age-related decline in executive function,^43^ and that the onset of dementia is often characterized by early impairment of executive function.^44^ Overall, our findings indicate that although *BDNF* Val homozygotes show normal positive associations between CR and executive function, *BDNF* Met carriers have a reduced influence of CR on executive function.

One explanation for the significant moderation of CR by *BDNF* Val66Met lies within differences in biochemical responses to cognitively stimulating environments. Namely, individuals with *BDNF* Met alleles may receive a lesser impact on neurological function of exposure to environmental activities that contribute to CR. Previous research has demonstrated that long-term exposure to cognitively stimulating environments is associated with a widespread increase in BDNF expression in the brain,^45^ but *BDNF* Met carriers have both reduced activity-dependent secretion of BDNF^22^ and impaired synaptic capacity for long-term potentiation/depression.^46^ Consequently, for two individuals exposed to the same level of extrinsic environmental stimulation, the individual who carries the *BDNF* Met polymorphism exhibits reduced synaptic plasticity and neuronal restructuring subsequent to environmental stimulation and consequently displays lowered functional capacity due to the negative intrinsic effect of BDNF Met polymorphism on development of CR. Studies examining the influence of environmental enrichment in rodents report that environmental enrichment only confers benefits in the presence of BDNF protein. For example, environment-induced hippocampal neurogenesis occurs only in the presence of BDNF^47^ and upregulation of BDNF by stimulating environments promotes neural plasticity.^48^

The results of the present study have several implications. First, as higher CR was associated with higher overall cognitive function, CR exerts an impact on healthy cognitive function, independent of dementia. Although the cross-sectional analyses employed in the present study do not allow comment on the relationship between CR and rate of age-related cognitive decline, the results indicate that, at the very least, CR may benefit cognitive function at older age due to the persistent effects of improved cognitive function. Second, the results of the present study demonstrate that a genetic factor moderates the effect that CR has on cognitive function. Specifically, *BDNF* Met carriers showed a reduced relationship between CR and executive function when compared with noncarriers. Although this effect was found within a relatively small sample size, this result provides initial evidence that *BDNF* Met carriers may have a partial ‘disconnect' between CR and cognitive function, and future longitudinal analyses will examine whether *BDNF* Val66Met mediates environmentally generated protection from dementia. In relation to noncarriers, the identification of persons with very high resistance to the functional consequences of brain pathology may provide a cohort for the development of future epigenetic strategies to reduce dementia in the face of AD disease progression.

## Figures and Tables

**Table 1:** Descriptive statistics for the study population stratified by *APOE* and *BDNF* genotypes

*Characteristic*	N	*APOE status*			*BDNF status*		
		*ɛ4-*	*ɛ4+*	P	*Met-*	*Met+*	P
*Demographics*							
Age (years)	422	62.31 (6.83)	61.67 (6.69)	0.53	62.16 (6.85)	61.99 (6.68)	0.81
Gender (male/female %)	422	31.9/68.1	35.8/64.2		31.6/68.4	36.4/63.6	
Genotype (%)	422	67.5	32.5		66.8	33.2	
							
*Screening*							
DRS-2 AEMSS	422	11.93 (2.12)	12.16 (2.03)	0.56	12.04 (2.10)	11.93 (2.08)	0.61
HADS anxiety	421	5.21 (3.12)	5.35 (2.97)	0.39	5.35 (3.19)	5.07 (2.80)	0.38
HADS depression	421	2.36 (2.14)	2.58 (2.34)	0.24	2.42 (2.26)	2.45 (2.10)	0.91
							
*Cognitive reserve*^28^							
WTAR Est. FSIQ	421	112.57 (5.39)	112.16 (5.94)	0.86	112.40 (5.49)	112.51 (5.76)	0.85
Prior education (years)	422	14.13 (2.81)	13.61 (2.59)	0.19	13.73 (2.72)	14.44 (2.75)	0.01
LEQ young adulthood specific	421	16.19 (7.93)	15.51 (7.65)	0.41	15.56 (7.61)	16.78 (8.25)	0.13
LEQ young adulthood nonspecific	421	24.71 (5.54)	24.93 (5.11)	0.19	24.42 (5.45)	25.52 (5.23)	0.05
LEQ midlife specific	420	19.07 (5.01)	19.06 (4.93)	0.45	19.09 (4.82)	19.02 (5.28)	0.90
LEQ midlife nonspecific	420	24.37 (5.55)	24.36 (5.60)	0.46	24.08 (5.72)	24.93 (5.20)	0.14
LEQ continuing education bonus	418	10.22 (8.79)	9.38 (8.01)	0.46	9.95 (8.45)	9.94 (8.75)	0.99

Abbreviations: AEMSS, age- and education-corrected Mayo Older American normative Studies (MOANS) scaled score; *APOE*, apolipoprotein E; *BDNF*, brain-derived neurotrophic factor; DRS-2, Dementia Rating Scale-2; FSIQ, full-scale intelligence quotient; HADS, Hospital Anxiety and Depression Scale; LEQ, Lifetime of Experiences Questionnaire; WTAR, Wechsler Test of Adult Reading.

Note: data represented are mean values (s.d.) for continuous variables and proportions for categorical variable. ɛ4−/Met−=participants not carrying the *APOE* ɛ4/*BDNF* Met alleles; ɛ4+/Met+=participants carrying at least one copy of the *APOE* ɛ4/*BDNF* Met alleles.

**Table 2:** Factor analysis results for composite cognitive domain variables

*Cognitive domain*	*Initial eigenvalue*	*Variable*	N	*Mean*	*s.d.*	*Loading*
Episodic memory	2.50 (62.46%)	RAVLT 1-5 total	407	52.96	8.92	0.76
		LM I immediate recall total	407	48.43	8.07	0.89
		LM II delayed recall total	407	30.29	6.28	0.86
		PAL first trial memory score	407	18.35	3.46	0.63
Working memory	2.04 (50.93%)	Digit span	407	18.63	3.90	0.76
		Letter–number sequencing	407	11.59	2.40	0.79
		SWM between errors	407	25.36	18.64	−0.63
		SSP length	407	5.75	1.20	0.66
Executive function	1.80 (59.83%)	Stroop trial C	403	26.26	7.63	0.76
		RVP A′	403	0.91	0.05	−0.81
		TMT trial B	403	59.09	18.82	0.75
Language processing	1.88 (62.81%)	WAIS vocabulary	421	56.62	6.23	0.88
		WAIS comprehension	421	26.21	3.33	0.77
		Boston naming test	421	57.49	3.42	0.72

Abbreviations: LM, logical memory; RAVLT, Rey auditory verbal learning test; RVP, rapid visual processing; SSP, spatial span; SWM, spatial working memory; TMT, trail making test; WAIS, Wechsler Adult Intelligence Scale.

Note: data in parentheses represent the proportion of variance (%) explained by the resulting factor.

**Table 3:** Results of the regression analyses for cognitive domain data

*Cognitive domain*	*Predictor*	N	β	*s.e.*	t	P	R^*2*^ *change*	F
Episodic memory	Age	401	−0.04	0.01	−6.25	**<0.01**		
	CR	401	0.05	0.02	2.56	**0.01**		
	Gender	401	0.53	0.10	5.40	**<0.001**		
	APOE	401	−0.06	0.10	−0.57	0.57		
	BDNF	401	−0.01	0.10	−0.09	0.93		
	CR × APOE	401	−0.01	0.04	−0.32	0.75	0.00	0.10
	CR × BDNF	401	−0.06	0.04	−1.40	0.16	0.00	1.97
Working memory	Age	401	−0.05	0.01	−7.18	**<0.01**		
	CR	401	0.07	0.02	3.26	**<0.01**		
	Gender	401	−0.10	0.10	−1.03	0.31		
	APOE	401	0.06	0.10	0.64	0.52		
	BDNF	401	−0.12	0.10	−1.19	0.24		
	CR × APOE	401	0.04	0.04	0.99	0.33	0.00	0.97
	CR × BDNF	401	0.04	0.04	0.98	0.33	0.00	0.96
Executive function	Age	397	−0.06	0.01	−9.25	**<0.01**		
	CR	397	0.11	0.02	5.40	**<0.01**		
	Gender	397	0.19	0.10	2.01	**0.045**		
	APOE	397	0.13	0.10	1.36	0.21		
	BDNF	397	−0.00	0.09	−0.05	0.96		
	CR × APOE	397	0.02	0.04	0.41	0.68	0.00	0.17
	CR × BDNF	397	−0.08	0.04	−1.97	**0.049**	0.01	3.88
Language processing	Age	415	0.00	0.01	0.36	0.62		
	CR	415	0.16	0.02	8.00	**<0.01**		
	Gender	415	−0.16	0.10	−1.67	0.10		
	APOE	415	0.08	0.10	0.79	0.43		
	BDNF	415	−0.07	0.10	−0.78	0.44		
	CR × APOE	415	0.03	0.04	0.60	0.55	0.00	0.36
	CR × BDNF	415	−0.07	0.04	−1.35	0.18	0.00	1.82

Abbreviations: APOE, apolipoprotein E; BDNF, brain-derived neurotrophic factor; CR, cognitive reserve.

Note: APOE and BDNF main and interaction effects were tested in separate models.

Bold values are statistically significant values with *P*<0.05.

## Notes

Associate Professor MJ Summers reports personal fees from Eli Lilly (Australia) Pty Ltd and grants from Novotech Pty Ltd, outside the submitted work. The remaining authors declare no conflict of interest.
